# Seminiferous tubule transfection in vitro to define post-meiotic gene regulation

**DOI:** 10.1186/1477-7827-7-67

**Published:** 2009-06-29

**Authors:** Sandra Danner, Christiane Kirchhoff, Richard Ivell

**Affiliations:** 1 Department of Andrology, University Clinic Hamburg-Eppendorf, 20246 Hamburg, Germany; 2 Research Centre for Reproductive Health, School of Molecular and Biomedical Science, University of Adelaide, Adelaide, SA 5005, Australia; 3 Fraunhofer Institute of Marine Biotechnology, 23562 Luebeck, Germany

## Abstract

**Background:**

Post-meiotically expressed genes in the testis are essential for the proper progression of spermatogenesis, and yet, aside from the construction of individual transgenic mice using specific promoters to drive reporter plasmids, there are only very limited possibilities for relevant and quantitative analysis of gene promoters. This is due to the special nature of post-meiotic haploid cells, which to date are not represented in any appropriate cell-lines. This article reports the development of novel methodology using isolated and cultured rat seminiferous tubules in a multiwell format, into which promoter-reporter constructs can be introduced by a combination of microinjection and electroporation.

**Methods:**

Culture conditions were developed which allowed the continued incubation of isolated rat seminiferous tubules for up to 48 h without obvious cell death and loss of post-meiotic cells. Transfection of intact seminiferous tubules by microinjection and electroporation was optimized to achieve high expression efficiencies of control plasmids, using either fluorescent protein or luciferase as reporters, thereby allowing both morphological as well as quantitative assessment.

**Results:**

Successful transfection was achieved into all cell types except for mature spermatozoa. However, there appeared to be only limited cell-type specificity for the promoters used, even though these had appeared to be specific when used in transgenic animals.

**Conclusion:**

We have devised a methodology which allows relatively high throughput analysis of post-meiotic gene promoters into primary cells of intact seminiferous tubules. An apparent lack of cell-type specificity suggests that the gene fragments used do not contain sufficient targeting information, or that the transient episomal expression of the constructs does not encourage appropriate expression specificity. The results also highlight the doubtful interpretation of many studies using heterologous transfection systems to analyse post-meiotically expressed genes.

## Background

In contrast to earlier assumptions, it is now evident that a large number of genes are expressed in meiotic and post-meiotic, haploid male germ cells, in late spermatocytes and spermatids [1]. This represents a special and discrete phase of transcription between the constraints of meiosis and the final replacement of most histones by the highly compact structure of transition proteins and subsequently protamines. At present there are only a few limited procedures available to examine the molecular details of gene regulation for such post-meiotically expressed genes, due to the lack of any suitable cell-lines for this stage of spermatogenesis. The majority of studies have employed heterologous transfection of promoter-reporter constructs into diploid somatic cells with co-transfection of expression constructs for various suspected transcription factors. This approach has the obvious tautological limitation that only factors can be characterized which are *a priori *suspected of being involved [2-5]. Hecht and colleagues attempted to overcome this problem by using an *in vitro *transcription assay comprising the promoter of the gene of interest linked to a G-free cassette, and using nuclear extracts from mature testes as the source of transcription factors [6]. Whilst providing useful information, this method lacks the discrimination of using transcription factors from specific cell types, and has proven difficult to reproduce in many laboratories. Conventional gain-of-function transgenesis has also been used to assess promoter specificity for post-meiotic genes [7-10]. However, this approach is largely limited to mice, and the fact that only a single construct can be used per individual animal has severely restricted both statistical analysis and a more detailed molecular dissection of promoter regions. Another procedure, which has permitted the analysis of post-meiotic promoters, is to transfect isolated spermatogonia *in vitro*, and then to transplant these into the testes of prepubertal or azoospermic animals [11]. Again, however, this method is restricted by the number of animals needed for reliable statistical analysis of individual constructs, by the inherent difficulty of germ cell transplantation itself, and the paucity of germ cells maturing through to post-meiotic stages. Finally, there are reports of direct *in vivo *transfection of gene constructs into the exposed seminiferous tubules of rodent testes, again with the limitation that a single testis is required for each construct, and that there are no appropriate means of quantification of the specific gene expression [12-16].

In order to redress this obvious methodological deficit, we have developed a procedure using explanted seminiferous tubules from rats, transfecting these with promoter-reporter constructs *in vitro *in a microtiter plate format, followed by short-term culture and quantitation. This new method allows many constructs to be characterized using tissues from a single animal, offers assessment of cell-type specificity, and provides a biologically relevant environment relatively free of the artefacts caused by using heterologous systems.

## Methods

### Preparation and culture of rat seminiferous tubules

All chemicals used were from Sigma-Aldrich (Deisenhofen, Germany), unless otherwise stated. Adult (7–8 months) male Wistar rats, which had been maintained under a 12 h/12 h light/dark regime with food and water *ad libitum*, were used for all experiments. Animals were killed by excessive CO_2 _anaesthesia, and testes immediately removed and further processed under sterile conditions. The *tunica albuginea *was slit to release seminiferous tubules, which were carefully teased apart, cut into approximately 10–15 mm lengths, transferred into a Petri dish containing TKM-medium (modified Tres – Kierszenbaum [17] medium: Minimal Essential Medium (without L-glutamine and phenol red; Gibco-Invitrogen; Karlsruhe, Germany), containing 5 μg/ml insulin (Invitrogen; Karlsruhe, Germany), 5 μg/ml human apo-transferrrin, 10 ng/ml epidermal growth factor (Upstate Biotechnology; Lake Placid, CA), 10 ng/ml insulin-like growth factor 1 (Upstate Biotechnology), 10 ng/ml human growth hormone (Eli Lilly; Bad Homburg, Germany), 10 ng/ml recombinant human follicle stimulating hormone (National Hormone and Peptide Program, Harbor – UCLA Medical Center, Torrance, CA), 5 μM retinol, 10 nM testosterone, 10 nM dihydrotestosterone, 4 mM L-glutamine, 1 mM sodium pyruvate, 0.1 mM non-essential amino acids (Gibco-Invitrogen), 100 U/ml penicillin, 100 μg/ml streptomycin) and then held in a tissue incubator at 32C (approximate scrotal temperature) and 5%CO_2_. All subsequent cultures were in 24-well plates containing 0.4 ml TKM medium per well.

**Figure 1:**
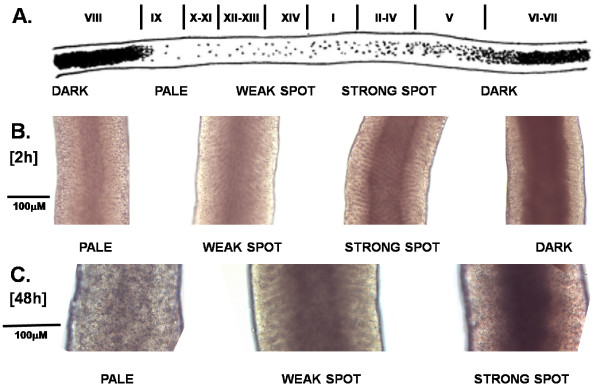
**Transillumination of rat seminiferous tubules to indicate the different stages chosen for microinjection and electroporation (after ref **[18]**)**. A. Diagram to show the visual correspondence between the different stages of spermatogenesis (roman numerals) and the terminology (dark, pale, weak spot, strong spot) used here. B. Transilluminated tubule sections after 2 hours of culture under basal conditions, without any further treatment. C. As in B, but after 48 h incubation.

Prior to culture in 24-well plates, tubules were sorted according to their transillumination pattern, following the scheme of Cheng and Mruk [18] (see Fig. 1). Initial analysis in order to check culture conditions made use of (a) gross morphology by transillumination, (b) microscopic analysis of frozen sections staining both with hematoxylin/eosin or using the fluorescent markers DAPI (for cell nuclei) and AlexaFluor-568 labelled PNA (peanut agglutinin; for labelling the acrosome of post-meiotic sperm), (c) immunohistochemistry also using immunofluorescence of frozen sections for the post-meiotic cytoplasmic marker protein endozepine-like peptide (ELP, also called Dbil5) [19,20].

Where using fluorescently labelled probes, cultured seminiferous tubules were fixed for 1 h in phosphate-buffered saline (PBS) containing 4% paraformaldehyde and 10% sucrose. Three to 4 tubules in similar orientation were then frozen together in Tissue-Tek medium (Sakura Finetek; Zoeterwoude, Netherlands) at -20C and stored at -80C. Sections of ca. 15 μM were cut, transferred to *SuperFrost *Plus slides (Menzel-Glaeser; Braunschweig, Germany), and stored at -80C until use. Sections were then air-dried for 1 h and then rehydrated in PBS containing 10 mM CaCl_2 _and 5 mM MgCl_2_. Sections were fluorescently labelled using PBS containing 10 mg/ml AlexaFluor 568 labelled PNA (Molecular Probes; Eugene, OR), 1 μg/ml DAPI (4',6-diaminodino-2-phenylindol; Merck; Darmstadt, Germany) or 2 μM TO-PRO-3-iodide (Molecular Probes) for 30 minutes in a moist chamber, rinsed twice for 5 mins in PBS, and then embedded in Fluoromount G (Southern Biotech; Birmingham, AL), prior to examination using a confocal or conventional fluorescence microscope (Leica TCS SL or Nikon Diaphot) as indicated. For immunohistochemistry, sections were rehydrated in PBS, followed by incubation in PBS containing 5% normal goat serum (NGS) for 30 min at room temperature, then in PBS containing 2% NGS plus 500:1 diluted rabbit polyclonal anti-ELP antiserum [20] overnight also at room temperature. Sections were then washed 3 times in PBS followed by incubation for 30 mins at room temperature with the secondary antibody (Cy3-labelled goat anti-rabbit IgG; Jackson ImmunoResearch; Newmarket, UK) diluted 100:1 in PBS with 2% NGS, also containing 1 μg/ml DAPI. After a further three washes in PBS, sections were mounted and analysed as above.

### Preparation of DNA constructs

For preliminary experiments to optimize and evaluate transfection and the expression of the transfected genes, the following vectors were used: pEGFP-N1 and pEYFP-N1 (both from BD Biosciences; Heidelberg, Germany) use the Enhanced Green Fluorescent Protein (EGFP) or Enhanced Yellow Fluorescent Protein (EYFP), respectively, driven from a CMV viral promoter. Also used was the pGL3-Control vector (pGL3-C; Promega; Mannheim, Germany) wherein the luciferase gene is driven from an SV40 promoter, using the promoterless pGL3-Basic vector (pGL3-B) as negative control. In order to test the specificity of different promoter regions from testis-expressed genes, PCR was used, employing gene-specific oligonucleotide primers (Table 1) to amplify defined regions of rat genomic DNA as indicated (Table 2). Resulting PCR fragments were ligated into the vector pDrive (Qiagen; Hilden, Germany) and sequenced, prior to restriction digestion and subcloning into the expression vector EYFP-N1 via the Ase1 restriction enzyme site incorporated into the forward primers of each PCR product, and the multiple cloning site of the pDrive vector, thereby replacing the CMV promoter of the original EYFP-N1 vector by the gene-specific promoter fragment. Alternatively, promoter fragments were cloned into the corresponding sites of the pGL3-C luciferase vector. All final constructs used were verified again by sequencing. For the PCR reactions from rat genomic DNA, 10 pmol of each oligonucleotide primer were used with 0.1 μg of rat genomic DNA as template together with 1.25 U of *ExTaq *(Takara Bio; Saint Germain-en-Laye, France) DNA polymerase in a total reaction mix of 50 μl. The PCR reactions comprised 35 cycles of denaturation (30 sec at 95C), primer annealing (30 sec at 55C to 62C; see Table 2), and synthesis (1 min at 72C), followed by a final elongation step at 72C for 5 min. The dimensions of the gene-specific promoter fragments for proacrosin [7], protamine 1 [8] and SP-10 [21] were determined based on the published results of using such promoters to drive tissue-specific expression in transgenic mice. For ELP, the fragments were based on our previous *in vitro *study of the mouse gene [5].

**Table 1:** Oligonucleotide sequences used for the cloning of diverse gene promoter fragments from rat genomic DNA.

**Promotor**	**Oligonucleotide sequence 5'-3'**
ratProtamine1_Ase_*forward*	TAT TAA TGT CTA GTA ATG TCC AAC AGC
ratProtamine1_*reverse*	AAC CTG TGA GCA GGT GGA ATT TTG
ratProacrosin_Ase_*forward*	TAT TAA TGG GTA GGA GCA TTC TCA TCT CGT
ratProacrosin_*reverse*	CAG ATC TGC CTG CAA GCT GTG ACC TCA CAA
ratSP-10_Ase_*forward*	TAT TAA TCC TCC AAT CTT AGG ACT AAC CTC
ratSP-10_*reverse*	TGG CAC ACT CAA GAG CTG AGA AGA AAC
ratELP10_Ase_*forward*	TAT TAA TGC AGG GTG TCA ACT AG
ratELP290_Ase_*forward*	TAT TAA TGT GCC ATC TCA GGC TGC
ratELP290_Bgl_*forward*	TAA GAT CTT CGT GCC ATC TCA GGC
ratELP-GCNF_Bgl_*forward*	TAA GAT CTT CAT TCG CTC GCG G
ratELP980_*reverse*	TTG TTG GAA AGG AGT ACG CGT G
ratELP1500_*reverse*	TAT ACC AGA AGC CGT GCC TCT G

**Table 2:** Summary of the amplified promoter sequences, the oligonucleotide PCR primers used, and their respective annealing temperatures.

**Gene promoter**	**Ref**	**Sequence**	**PCR primers**	**PCR product**	**Annealing temperature**
SP10	[9,21]	-403 to +28	ratSP10_Ase_f ratSP10_r	437 bp	55°C
Proacrosin	[7,26]	-263 to +48	ratProacro_Ase_f ratProacro_r	818 bp	62°C
Protamine 1	[8]	-556 to +30	ratPrm1_Ase_f ratPrm1_r	593 bp	55°C
Endozepine-like peptide (ELP)	[5,19]	-652 to +51	ratELP290_Ase_f ratELP980_r	708 bp	55°C
		-957 to +617	ratELP10_Ase_f rat ELP1500_r	1571 bp	62°C
		-957 to +51	ratELP10_Ase_f ratELP980_r	1005 bp	60°C
		-652 to +617	ratELP290_Ase_f ratELP1500_r	1274 bp	60°C
		-652 to +51	ratELP290Bgl_f ratELP980_r	708 bp	60°C
		-652 to +617	ratELP290_Bgl_f ratELP1500_r	1274 bp	62°C
		-380 to +617	ratELP-GCNF_Bgl_f ratELP1500_r	1003 bp	60°C

Oligonucleotide primers (see Table 1) were used in the combinations indicated with rat genomic DNA as template to generate PCR products of the correct size. The sequence parameters are given relative to the transcription start site (+1) determined from the corresponding homologous mouse sequences, given in the references. PCR conditions are given in the text.

### Transfection and analysis of introduced DNA constructs

The procedure used to introduce DNA expression vectors into explanted seminiferous tubule fragments was largely based on methods developed for gene therapy *in vivo *e.g. [12,13]. After considerable preliminary experimentation, varying a wide range of possible parameters, the following protocol was considered optimal for EYFP-N1 or EGFP-N1 constructs. DNA solution at a concentration of 1 μg/μl was microinjected laterally into an individual tubule fragment that had been arranged lengthwise on a microscope slide. Microinjection used an Eppendorf Transjektor 5426 attached to an Eppendorf Micromanipulator 5171, both mounted onto the stage of a Zeiss Axiophot inverse microscope, providing a high degree of sensitivity and control over the microinjection process. The DNA was injected using a glass microcapillary whereby the tip had been deliberately fractured to provide a cutting surface allowing good penetration of the tubule *lamina propria*, and thus release of DNA into the tubule lumen. DNA volume was regulated to fill ca. 30% of the tubule length. This represents an excess, since the amount transfected is determined not by the volume of DNA but by the lateral dimensions of the *Tweezertrodes *used for electroporation (see below). Following microinjection, tubule fragments are transferred to a 24-well plate containing 0.4 ml TKM medium per well. *Tweezertrodes *(Cyto-Pulse Inc.; Glen Burnie, MD) are inserted into a well, straddling a tubule fragment, and transfection achieved by electroporation using an electrode gap of 4 mm, a pulse number of 10, an individual pulse strength of 20 V, a pulse length of 20 ms, and a pulse interval of 0.5 s, using a Cyto-Pulse PA-4000S square wave electroporation system. These parameters were determined following extensive variation and optimization in preliminary experiments. Transfected tubules were then transferred to an incubator at 32C/5% CO_2 _and examined at various intervals up to 48 h.

Seminiferous tubules were analysed for the expression of EYFP and EGFP at the indicated time intervals by direct fluorescence following excitation at 488 nm. Where luciferase expression constructs were used, protein lysates were prepared from tubules by first shock-freezing tubules in liquid nitrogen, pulverizing these in a small pestle and mortar, then dissolving in 150 μl of Reporter Lysis Buffer (Luciferase Assay System; Promega). After brief (2 min) centrifugation at 13000 rpm, 20 μl of the lysate was supplemented with 100 μl of Luciferase Substrate Solution (Luciferase Assay System; Promega), with measurement for 10 sec in a single-tube luminometer (Berthold; Bad Wildbad, Germany).

In order to assess the effect of culture and transfection conditions on the viability of cells in the explanted seminiferous tubules the *LIVE/DEAD Viability/Cytotoxicity Kit *(Molecular Probes) was used. The assay measures both the integrity of the plasma membrane and intracellular esterase activity using a mixture of Calcein-AM and Ethidium-Homodimer-1 (EthD-1), which differ in their membrane permeability. Calcein-AM is able to penetrate most cell membranes and is then cleaved inside cells by the esterase activity of living cells to form fluorescent Calcein (excitation 495 nm/emission 515 nm). Eth-D1 only enters damaged cells, where it complexes with DNA to yield a high nuclear fluorescence in the red spectrum (excitation 495 nm/emission 635 nm). Tubules are incubated in a mixture of EthD-1 (4 μM) and Calcein-AM (2 μM) in PBS for 30 mins at room temperature with subsequent analysis by confocal microscopy. Additionally, tubules were assessed following culture and/or transfection by TUNEL (*Terminal dUTP nick end labelling*) as a measure of apoptotic cell death, using the *In situ Cell Death Detection Kit *(TMR red; Roche Diagnostics; Mannheim, Germany) on tubule cryosections exactly following the manufacturer's instructions. This assay can lead to spurious results when used on testicular tissue because of the natural phagocytic activity of the Sertoli cells. This can be avoided by masking the "eat me" signals of apoptotic cells with Annexin V (10 μM; Apotech; Epalinges, Switzerland), which is microinjected into the seminiferous tubules simultaneously with the DNA constructs and blocks phagocytosis.

### Sertoli cell culture, transfection and analysis

The mouse SK11 Sertoli cell-line [22] was additionally used to test the activity and specificity of some of the promoter-reporter constructs. These adherent cells were grown at 32C/5% CO_2 _in 250 ml flasks in DMEM (Dulbecco's Minimal Essential Medium – High Glucose; Gibco-Invitrogen) containing 10% fetal calf serum, 100 U/ml penicillin, and 100 μg/ml streptomycin, with passaging every 3 to 4 days. For transfection, cells were transferred to either 24-well plates (for luciferase assay) or 4-well chamber slides (for microscopic fluorescence assessment), and cultured overnight at 32C/5%CO_2_. Medium was then replaced by DMEM lacking additives, and 1 mg per well of DNA transfected using the Lipofectamine 2000 reagent (Invitrogen) according to the manufacturer's instructions. After transfection, cells were incubated for 5 h as above, then medium was replaced by DMEM plus 10% FCS, and incubation continued for a further 24 h until measurement of reporter gene expression. For assessment of luciferase activity the Luciferase Assay System (Promega) was used as already described.

## Results

### Establishment of culture conditions

**Figure 2:**
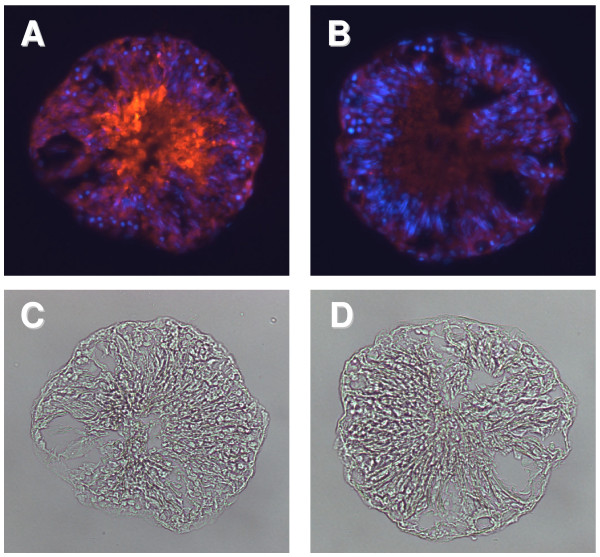
**Cryostat sections of rat seminiferous tubules cultured for 48 h without further treatment, to indicate the integrity of all spermatogenic stages after such incubation**. A. Indirect Cy3 immunofluorescence for ELP protein (red); all nuclei are stained using DAPI (blue). Note the persistence of large numbers of positively ELP-stained elongated spermatids, even after 48 h of culture. B. As in A, but using the pre-immune serum as negative control. C and D are the brightfield images of the sections in A and B, respectively.

In order to provide the best possible cellular environment for appropriate gene expression, culture conditions were optimized so as to maintain maximal tubule integrity in terms of cell representation and form over a period of 48 hours for tubules representing all four gross stages of spermatogenesis (Fig. 1). In order to verify the persistent presence of late post-meiotic germ cells, specific antibodies for the spermatid marker endozepine-like peptide (ELP) [19] were used (Fig. 2). Whilst it is unavoidable that there is some loss of structural integrity of the tubules with culture, as has been reported previously [23], the quantitative maintenance of ELP-immunostaining implies that there is no selective loss of post-meiotic germ cells in this culture system, and indirectly that the Sertoli cells are also providing appropriate supportive function.

### Optimization of transfection parameters

**Figure 3:**
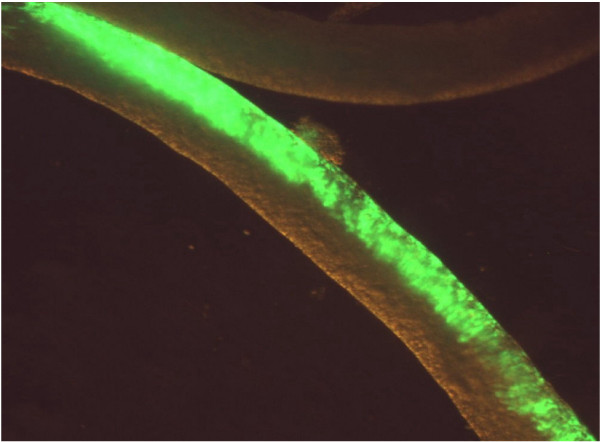
**Rat seminiferous tubule 24 h following optimal transfection with the reporter plasmid EGFP-N1 driven from a constitutive CMV promoter**.

**Figure 4:**
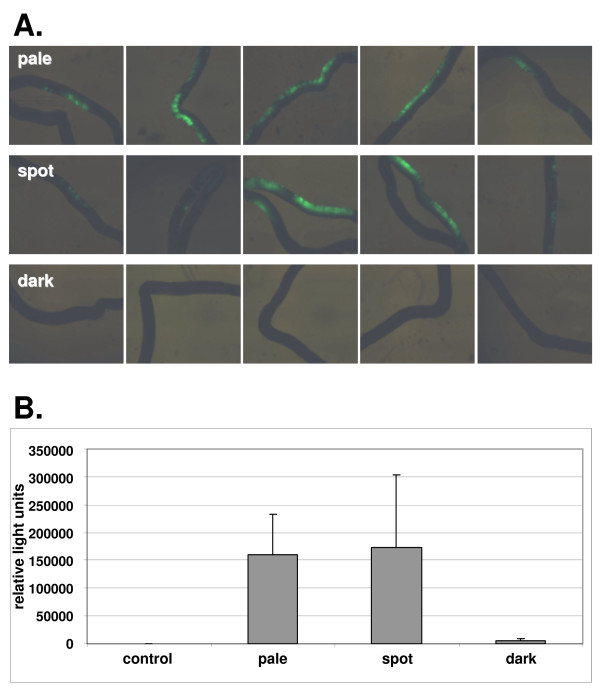
**A. A comparison of the transfection efficiency for the control plasmid EYFP-N1 of different stages of spermatogenesis (judged by transillumination) after 24 h incubation**. Each row represents typical individual tubules for each of the indicated stages. B. A similar comparison but using the reporter plasmid pGL3-C, expressing the luciferase gene, and quantifying the specific luminescence after 24 h incubation from 10 tubules of each stage. For the control, tubules were transfected with the promoterless control plasmid pGL3-B, in order to determine any background endogenous luminescence.

In order to optimize the parameters for gene transfection, the constructs EGFP-N1 or EYFP-N1 were used, always at a constant concentration of 1 μg/μl in DNA injection medium (see Materials and Methods). For electroporation, electrode distance (mm), pulse number, pulse strength (V), pulse length (ms), and pulse interval (s) were all varied and transfection efficiency estimated from the percent of positively transfected tubuli. Optimal transfection (Fig. 3) of up to 80% was thereby found for an electrode distance of 4 mm, applying 10 pulses each of 20 V for 20 ms each with 1 s interval between pulses. It should be noted that transfection always occurs on one side of a tubule only (Fig. 3), with least efficient transfection likely to be in the mural cells due to the physical obstruction of the more luminal cell layers. In the course of this preliminary study, it was observed that some tubule sections consistently and stage-specifically yielded higher transfection efficiencies than others. Therefore, in a new experiment 10 tubules of each stage "pale", "spot", and "dark" (see Fig. 1) were transfected using the CMV-driven luciferase reporter construct pGL3 and incubated for 24 hours, before determining luciferase activity (Fig. 4). These results show that the presence of large numbers of mature spermatozoa at stages VI to VIII inhibited transfection.

**Figure 5:**
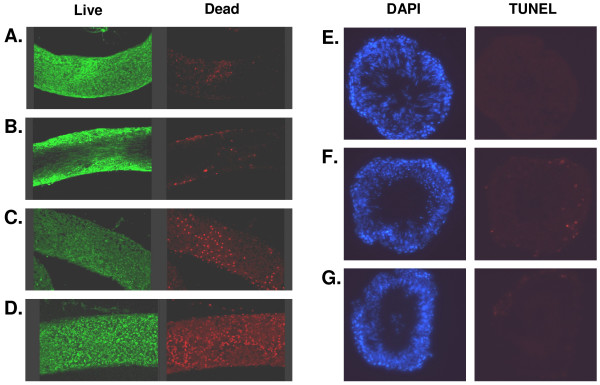
**Cell death induced by the microinjection and electroporation technique**. A-D. The *Live-Dead *assay in intact seminiferous tubules assessed by confocal microscopy projected from 14 superimposed sections, using calcein fluorescence of all living cells (green) compared to the ethidium homodimer fluorescence (red) of the dead cell nuclei. A. Basal conditions after 24 h incubation. B. Basal conditions after 48 h incubation. C. After 24 h incubation following DNA microinjection. D. After 24 h incubation following DNA microinjection and electroporation. Note only under these conditions is the evidence of significant cell death. E-G. TUNEL analysis of cryosections from incubated seminiferous tubules. All nuclei are labelled blue with DAPI. The TUNEL assay used tetramethyl rhodamine to label nuclei with nicked DNA (red). E. Tubule after 48 h incubation under basal conditions – no obvious DNA damage. F. Tubule after 48 h incubation following microinjection of 10 μM annexin V to block phagocytosis – some limited DNA damage in mural cells. G. Tubule after 48 h incubation following DNA injection – negligible DNA damage evident.

Finally, the optimal electroporation parameters were checked for their impact on cell survival and apoptosis by analysing transfected tubules using the *Live-Dead *assay, as well as by conventional TUNEL. The *Live-Dead *assay showed that DNA injection and electroporation, but not culture *per se*, led to some cell damage and death (Fig. 5A–D), but that this damage did not appear to be cell selective, and the majority of cells remained intact. Application of TUNEL in the presence and absence of Annexin V in order to arrest any phagocytotic activity indicated very few labelled cells, mostly at the tubule margins (presumably spermatogonia or early spermatocytes), and then only after 48 hours following the full procedure (Fig. 5E–G).

### Cell specificity of transfection

**Figure 6:**
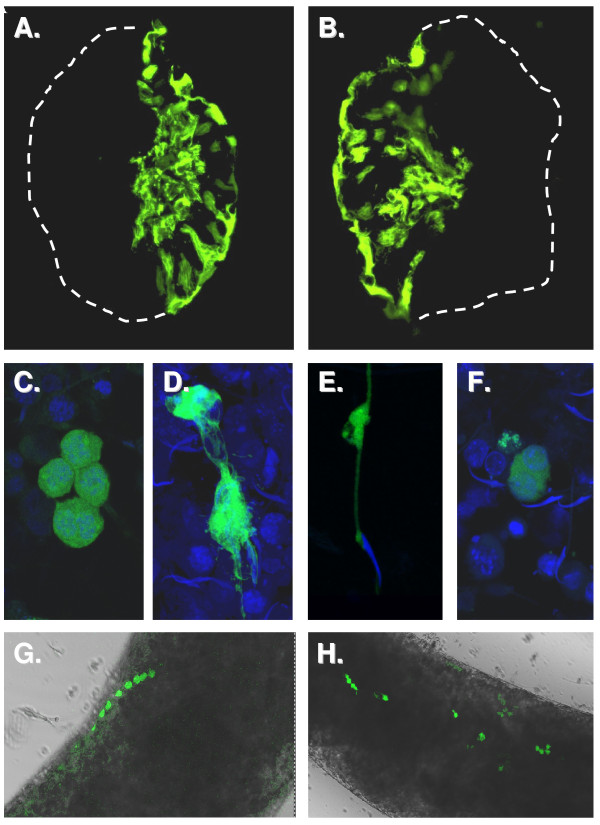
**A-B. Cryosections showing EYFP fluorescence in almost all cell types 24 h following microinjection and electroporation of the CMV-driven EYFP-N1 plasmid**. Note that only one half of the tubule is successfully electroporated. The tubule outline is shown by the white dashed line. C-F. EYFP fluorescence in different cell types 24 h following microinjection and electroporation of the CMV-driven EYFP-N1 with subsequent dissolution of the tubule and cytospin centrifugation of individual cells. Cell nuclei are labelled using TO-PRO. G-H. Direct fluorescence of EYFP in intact seminiferous tubules 24 h following microinjection and electroporation of the CMV-driven EYFP-N1 plasmid.

Further control experiments were undertaken to check that all cell types of the seminiferous epithelium were potentially transfectable, and that results would not be distorted by a cell selectivity of transfection. Using the CMV-driven EYFP reporter construct and confocal microscopy of isolated tubules indicated fluorescence in round spermatids or spermatocytes, and in probable Sertoli cells. This was confirmed by assessing EYFP fluorescence in cryosections of tubules (Fig. 6A, B), as well as in cytospin preparations of dispersed tubule cells (Fig. 6C–FB). Figs. 6G and 6H illustrate probable transfection of early round spermatid syncytia. These results show that transfection appears to be possible in all of the post-meiotic germ cell types, as well as in Sertoli cells.

### Expression of cell-specific promoter-reporter constructs

**Figure 7:**
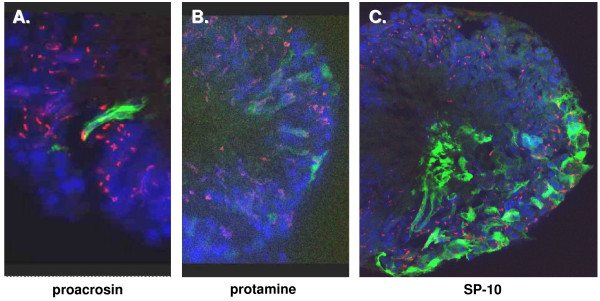
**EYFP expression (green) in seminiferous tubules 24 h after transfection with an EYFP reporter plasmid driven from (A) the proacrosin gene promoter, (B) the protamine gene promoter, and (C) the SP-10 gene promoter**. All nuclei are labelled with DAPI (blue), and mature sperm acrosomes are labelled using PNA (red).

Following the preliminary establishment of methodology, apparently well-characterized testis gene promoters were used to check cell- and stage-specificity of expression. All of these promoters had been shown to exhibit specific expression in transgenic mice. The promoters of the rodent proacrosin, protamine 1, and SP-10 genes were all shown to be active in seminiferous tubules. However, the pattern of expression did not always meet expectation. The proacrosin promoter appeared to be expressed in both round and elongated spermatids, as anticipated, but also in Sertoli cells (Fig. 7A). Weak fluorescence is also detectable for the protamine-1 promoter in elongated spermatids, as expected, although there appears also to be weak expression in some Sertoli cells (Fig. 7B). The SP-10 promoter offered the greatest surprise with considerable expression clearly in Sertoli cells (Fig. 7C), with additional fluorescence evident in some round cells, possibly spermatids. Because it has been reported that the normal EYFP protein may be toxic to some cells and thus distort its expression [24], additional experiments were carried out with the EYFP being targeted to mitochondria by an additional address sequence. The results, however, were similar with expression in both spermatids and Sertoli cells (not shown).

**Figure 8:**
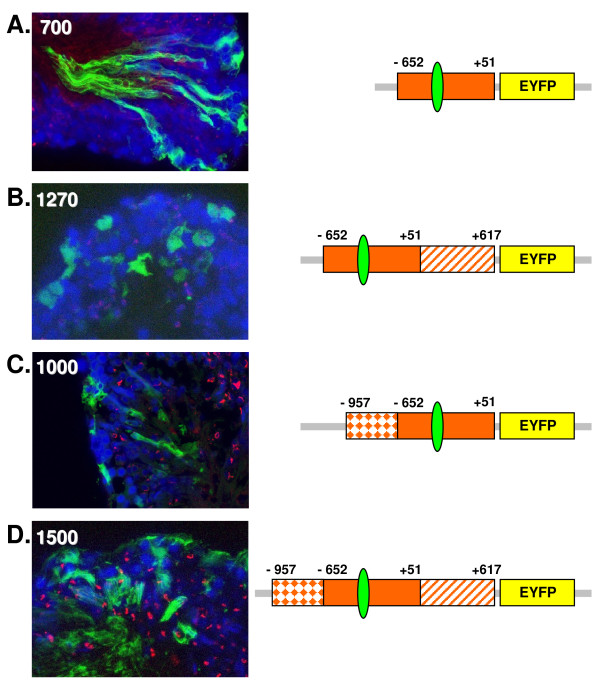
**As in Figure 7, but using a range of EYFP reporter gene constructs driven by different regions of the ELP gene promoter, as indicated on the right**. For details of the promoter, see ref [5]. The green ellipse indicates the position of the GCNF binding site, determined by in vitro methods [5]; the hatched box (+51 to +617) represents the only intron in the gene, which immediately precedes the methionine translational start codon, and which might contain regulatory information.

**Figure 9:**
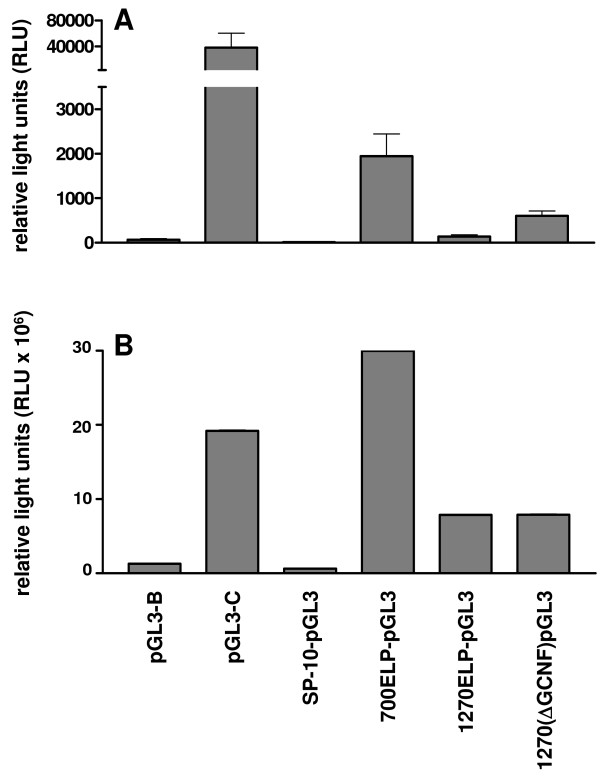
**A. Analysis of luciferase activity in isolated seminiferous tubules 24 h following transfection with different promoter-reporter constructs, as indicated in the Materials and Methods section**. **B**. Analysis of luciferase activity in the SK11 Sertoli cell line following transient transfection with the same promoter-reporter constructs as in A. Note that the error bars in panel B represent such small within-experiment variation that they are not easily visible in this figure.

Further experiments were also carried out using the recently defined promoter region of the ELP gene, which is expressed with high specificity in post-meiotic germ cells in rodents [5]. Consistently all constructs indicated expression both in Sertoli as well as in germ cells (Fig. 8). In order to check the cell specificity of these promoters further, similar promoter constructs but driving a luciferase reporter gene were additionally transfected into the mouse Sertoli cell line SK11 [22]. Like all of the ELP promoter constructs (Fig. 9B), also the proacrosin promoter indicated significant basal expression in the Sertoli cell line (not shown), though there was no evident expression for either the protamine-1 (not shown) or SP-10 promoters (Fig. 9B), suggesting that these may have retained a degree of cell specificity in this cell-line. A similar expression profile was observed using the same luciferase constructs transfected into seminiferous tubules and measuring the activity directly in tubule homogenates (Fig. 9A), implying that much of the ELP construct expression there is probably also in Sertoli cells. Since we had shown that a significant transcription factor controlling the expression of the ELP gene in mice is represented by a GCNF (germ cell nuclear factor) binding site in the promoter [5], and that this may be activated not only by GCNF but also be the factor SF-1 (steroidogenic factor 1), which is also present in Sertoli cells, a further construct was used in which this site had been deleted (1270(ΔGCNF)pGL3). Also this construct appeared to be expressed with equal efficiency in Sertoli cells (Fig. 9).

## Discussion

We describe here an assay system to evaluate the qualitative and quantitative attributes of specific testicular gene promoters. In particular, this system seems suitable to analyse the promoter regions of genes expressed in the natural context of post-meiotic germ cells, which until now could only be assessed through *in vitro *transcription assays, or through transgenesis or similar approaches. Optimal parameters were determined for the introduction of gene promoter-reporter constructs into isolated seminiferous tubules by microinjection followed by pulsed electroporation. The advantage of this method is that it potentially allows both a qualitative assessment of cell specificity of expression, by using reporter genes encoding enhanced fluorescent proteins, as well as a quantitative assessment using either these constructs or better a luciferase reporter construct. The method would also be amenable to using other quantifiable reporter genes, such as that for human growth hormone [25] or chloramphenicol acetyl transferase (CAT). The major advantage, however, is that different animal species can be used, and from a single organ many tubule pieces can be collected and arrayed in a microtiter plate format for analysis, allowing a large number of constructs with multiple repeats to be tested. Based on the expression patterns from both a general viral promoter-driven construct, as well from constructs using more specific promoters, it is evident that most germ cell types as well as Sertoli cells can be targeted. The obvious exception here is the mature spermatozoon, which within the "dark" tubule sections appears not to be able to incorporate expression constructs, or if transfection does occur to these cells, they are unable to express the construct through a lack of appropriate cellular machinery.

Although this preliminary study was intended only to establish working parameters and to develop proof of principle, it has already highlighted some very interesting observations. Namely, that several so-called specific promoter regions, do not appear to delineate the degree of cell specificity that had been expected from the literature. The 877 bp promoter sequence of the proacrosin gene had been shown in transgenic mice to target a CAT reporter specifically to spermatocytes and spermatids [7,26]. The same promoter in the present study appears to target both spermatids and Sertoli cells. Similarly, the promoters for the post-meiotically expressed genes SP-10 and protamine-1 had been shown to target specifically spermatids in transgenic mice [8-10,21] in a stage-specific manner, but additionally indicated promiscuous expression in Sertoli cells following *in vitro *transfection into seminiferous tubules. Application of these promoters for *in vivo *transfection into seminiferous tubules also indicated a similar stage-specific expression in haploid cells [12,13]. However, more recent studies using *in vivo *transfected seminiferous tubules and the gene promoters for the haploid-expressed genes for phosphoglycerate kinase 2 (Pgk2) [27] as well as for hst70 [14] also indicate a promiscuous expression of the reporter genes in Sertoli cells, in addition to germ cells. Our experiments here with the ELP gene promoter largely confirm these observations concerning lack of cell specificity.

There are several possible reasons for this Sertoli cell expression of supposedly germ cell specific gene promoters. Firstly, when compared to transgenic expression, episomally expressed genes may lack certain stringency criteria provided by the embedding of a gene in a conventional chromatin environment. This may be epigenetic in nature, or may arise from the existence of neighbouring isolator or silencer sequences in the genomic neighbourhood of the insertion sites, all of which are likely to be missing in episomally expressed genes. It is reported that the specificity of the SP-10 promoter is in part due to the presence of an isolator sequence within its natural genomic location [21]. A second possibility is that Sertoli cells are notoriously able to phagocytose damaged cells, particularly germ cells [28], and may thus express fluorescent protein by dint of having incorporated a germ cell expressing the reporter gene. We and others [15,29] have shown that the microinjection and electroporation procedure used, whilst having minimal effect on cell death nevertheless does indeed lead to an increase in apoptosis, as assessed by TUNEL. In future studies, it will be important to test the effect of using much larger regulatory DNA regions which include the proximal promoter within a larger natural context, as well as to test the effect of different blockers of apoptosis and phagocytosis on the apparent cell specificity of reporter gene expression. Furthermore, the addition of a cell-separation step to isolate different component cells of the seminiferous tubules prior to reporter quantification, as suggested in [29], might prove valuable. The apparent expression of the proacrosin and ELP promoters in the Sertoli cell line SK11 may be due in part to the illegitimate use of Sertoli cell transcription factors which may share DNA binding sites with germ cell factors, as well as to an absence of restricting silencer elements in the larger DNA context of the natural gene locus. In contrast, the lack of promiscuous expression of the protamine-1 and SP-10 promoters in the SK11 cells could mean that, unlike in the tubules, these Sertoli cells, being an immortalized cell-line growing under basal conditions, may simply not express the appropriate factors, which are produced by primary Sertoli cells growing in the more natural milieu of the seminiferous tubule with moderate hormonal stimulation.

## Conclusion

In conclusion, we have demonstrated that isolated seminiferous tubule fragments cultured *in vitro *for up to 2 days can be transfected with specific testicular promoter-reporter constructs yielding both qualitative and quantitative output. Further refinement of this method should lead to a valuable routine assay system for the evaluation especially of haploid expressed gene promoters, which to date cannot be assessed simply and accurately by any other procedure. This initial study has already highlighted the very important observation that several promoters, which from transgenic studies appear to be absolutely cell-type specific, lose some of that specificity when introduced by transient transfection.

## Competing interests

The authors declare that they have no competing interests.

## Authors' contributions

SD carried out most of the experimental work as part of her PhD thesis submitted to and accepted by the University of Hamburg. She contributed substantially to the conceptual development, planning and reporting of the experiments. CK was jointly responsible for overall supervision and coordination of the project. RI initiated, conceived, and jointly supervised the project, and was overall responsible for the interpretation and reporting of the results. All authors read and approved the final manuscript.
